# A Mixed-Methods Evaluation of Mainstreaming Mass Drug Administration for Schistosomiasis and Soil-Transmitted Helminthiasis in Four Districts of Nigeria

**DOI:** 10.4269/ajtmh.23-0600

**Published:** 2024-04-09

**Authors:** Emily Griswold, Abel Eigege, Emmanuel C. Emukah, Jayden Pace Gallagher, Jenna Coalson, Lindsay Rakers, Bulus Mancha, Okocha Ndudi, Paul Ugbadamu, Philomena Dikedi, Happiness Poko, Jacob Danboyi, Philemon Dagwa, Vincent Anighoro, Christiana Davou Gwong, Esther Otabor, Goodluck James Amayat, Regina Ese Unukopia, Emmanuel S. Miri, Gregory S. Noland

**Affiliations:** ^1^ The Carter Center, Atlanta, Georgia;; ^2^ The Carter Center, Jos, Nigeria;; ^3^ The Carter Center, Benin City, Nigeria;; ^4^ Edo State Primary Health Care Development Agency, Nigeria;; ^5^ Nasarawa State Ministry of Health, Nigeria;; ^6^ Plateau State Ministry of Health, Nigeria;; ^7^ Delta State Primary Health Care Development Agency, Nigeria;; ^8^ NTD Unit, Bassa LGA, Plateau State, Nigeria;; ^9^ NTD Unit, Egor LGA, Edo State, Nigeria;; ^10^ NTD Unit, Wamba LGA, Nasarawa State, Nigeria;; ^11^ NTD Unit, Ughelli South LGA, Delta State, Nigeria

## Abstract

In Nigeria, mass drug administration (MDA) for schistosomiasis (SCH) and soil-transmitted helminthiasis (STH) has often been coordinated with other programs that receive greater external funding. As these programs reach stop MDA milestones, SCH and STH programs will likely need to transition implementation, or “mainstream,” to domestic support. A mixed-methods study was conducted in four districts before (2021) and after (2022) mainstreaming to evaluate its impact on MDA coverage. Household surveys were done in 30 villages per district pre- and post-mainstreaming. All selected communities were eligible for STH treatment; around a third were eligible for SCH treatment. Mass drug administration was primarily conducted in schools. A total of 5,441 school-aged children were included in pre-mainstreaming and 5,789 were included in post-mainstreaming. Mass drug administration coverage was heterogeneous, but overall, mebendazole coverage declined nonsignificantly from 81% pre-mainstreaming to 76% post-mainstreaming (*P =* 0.09); praziquantel coverage declined significantly from 73% to 55% (*P =* 0.008). Coverage was significantly lower among unenrolled children or those reporting poor school attendance in nearly every survey. For the qualitative component, 173 interviews and 74 focus groups were conducted with diverse stakeholders. Respondents were deeply pessimistic about the future of MDA after mainstreaming and strongly supported a gradual transition to full government ownership. Participants formulated recommendations for effective mainstreaming: clear budget allocation by governments, robust and targeted training, trust building, and comprehensive advocacy. Although participants lacked confidence that SCH and STH programs could be sustained after reductions in external support, initial results indicate that MDA coverage can remain high 1 year into mainstreaming.

## INTRODUCTION

Nigeria is one of the countries with the highest burdens of schistosomiasis (SCH) and soil-transmitted helminthiasis (STH) in the world. Of the estimated 1.5 billion people around the world with STH, Nigeria is home to more than 48 million and is endemic for *Ascaris lumbricoides*, *Trichuris trichiura*, and both hookworm species: *Necator americanus* and *Ancylostoma duodenale*.^1^^,^^2^ More than 200 million people worldwide are believed to have SCH, and Nigeria has the world’s highest burden of these infections, specifically *Schistosoma mansoni* or *Schistosoma haematobium*.^3^^–^^5^ School-aged children (SAC) often carry the largest burden of SCH and STH and suffer significant morbidity, including malnourishment, anemia, and other harm.^6^

Schistosomiasis and STH join onchocerciasis and lymphatic filariasis as the four helminthic neglected tropical diseases (NTDs) for which preventive chemotherapy, or mass drug administration (MDA), is the core intervention recommended by the WHO.^7^ The WHO currently aims to reduce high-intensity SCH and STH infections by providing praziquantel and albendazole/mebendazole, respectively, to SAC. In Nigeria, these medicines are typically delivered to at-risk children living in endemic areas through school-based MDA but may be delivered through a community-based MDA platform, such as that for onchocerciasis and/or lymphatic filariasis.^8^ The albendazole component of lymphatic filariasis (LF) MDA can also double as STH treatment and often reaches more children than a school-based platform.^9^ Interventions against these diseases are administratively integrated within Nigeria, although implementation varies by context.^10^

Despite a recent change in WHO strategy toward elimination of transmission for SCH, this goal is unlikely to be reached without significant sanitation improvements. Thus, both SCH and STH treatments may need to be sustained indefinitely to control prevalence and intensity of infections.^11^^–^^13^ Increasingly, donors and implementing partners advocate “mainstreaming” these programs, that is, ceasing external support and domesticating all aspects of program implementation to each endemic country’s primary healthcare and education systems to ensure continuous operations, regardless of donor engagement. Furthermore, facilitating country ownership is a primary pillar of the global strategy for NTDs.^14^ Yet there is limited evidence on the viability of this model in sustaining SCH and STH treatment coverage targets after the withdrawal of external support.

The purpose of this study was to evaluate the process and impact on coverage of SCH and STH MDA in four districts of Nigeria after a transition to full domestic implementation. We undertook a mixed-methods evaluation of the mainstreaming process, measuring MDA coverage as well as investigating people’s perceptions of and reactions to the withdrawal of nongovernmental organization (NGO) support for MDA through key informant interviews (KIIs) and focus groups.

## MATERIALS AND METHODS

### Study setting.

The Carter Center (TCC), in consultation with state ministries of health, selected four districts in Nigeria in four states: Ughelli South in Delta State, Egor in Edo State, Wamba in Nasarawa State, and Bassa in Plateau State. All four were receiving assistance from TCC only for STH and SCH MDA, as they were either nonendemic or had stopped MDA for onchocerciasis and/or LF. These states have generally poor sanitation environments and varied yet persistent endemicity of SCH and STH.^15^^–^^19^ Although STH endemicity is ubiquitous in the study area, SCH endemicity is more heterogeneous. As such, STH MDA with mebendazole is conducted throughout each of the study districts, but SCH MDA with praziquantel is targeted only in endemic wards, the subdistrict administrative unit. Therefore, all selected communities were targeted to receive mebendazole, but only a portion were targeted for praziquantel distribution. Mass drug administration for SCH and/or STH is traditionally delivered in a single, integrated campaign to children at schools once per year in this context.

The pre-mainstreaming round of MDA, fully assisted by TCC, occurred in July 2021. Coverage surveys began in August 2021. At that time, it became clear that MDA had not yet finished in Egor District. The coverage survey in Egor was paused, and additional efforts were made to complete the MDA. A fresh sample was drawn, and the Egor survey was redone the following month. Only the results of this second survey are presented here as “pre-mainstreaming.” The “post-mainstreaming” surveys were done in July and August of 2022, also within 1 month of MDA. Interviews and focus groups occurred in July and August of 2021, April and May of 2022, and July and August of 2022.

### Mainstreaming decisions.

The Carter Center facilitated planning meetings at the beginning of the study to determine how mainstreaming would be conducted. These were attended by representatives from the health and education sectors. Other participants included representatives from the office of the executive governor and the budget office. Districts were not required to implement MDA in the same way. Attendees participated in activities to list all the steps needed to undertake MDA as well as the personnel involved. The representatives of each district then collectively determined how these responsibilities would be handled within their own district. These plans were adapted and finalized between the two rounds of MDA considered in this study. The delineation of duties for each district is described in Table 1. Of note, TCC continued supporting the shipment of drugs from the central warehouse in Lagos to the states by paying for the state pharmacists’ travel. Once drugs arrived at the states, Carter Center staff and vehicles distributed medicines to local government areas (LGAs) and thence to various schools and frontline health facilities. Finally, there were nationwide shortages of praziquantel during this study. The Carter Center redistributed some praziquantel from elsewhere in Plateau State to Edo and Delta for the purposes of this study.

**Table 1:** MDA responsibilities before and after mainstreaming, 2021–2022

Lead Responsibility	2021	2022			
Activities	All LGAs	Bassa	Wamba	Egor	Ughelli South
Advocacy	TCC	TCC	TCC	TCC, PHCDA	TCC, PHCDA
Drug logistics	TCC	TCC	TCC	TCC	TCC, PHCDA, MCH
Mobilization	TCC	SUBEB	SUBEB	PHCDA	PHCDA
Sensitization	TCC	LGA/SUBEB	LGA/SUBEB	PHCDA	PHCDA
Training	TCC	Not done	Not done	PHCDA	PHCDA
MDA	TCC	SBMC/PTA	SUBEB SBMC/PTA	PHCDA	PHCDA
Supervision and monitoring	TCC	Not done	Not done	PHCDA	PHCDA
Data collation and collection	TCC	SBMC/PTA	SBMC/PTA	PHCDA	PHCDA
Reporting	TCC	SBMC/PTA	SBMC/PTA	PHCDA	PHCDA

LGA = local government area (district); MCH = Maternal and Child Health; MDA = mass drug administration; PHCDA = Primary Health Care Development Agency; PTA = parent-teacher association; SBMC = school-based management committee; SUBEB = State Universal Basic Education Board; TCC = The Carter Center.

### Quantitative MDA coverage surveys pre- and post-mainstreaming.

To measure the impact of mainstreaming on SCH/STH MDA coverage, we undertook household-based coverage surveys of mebendazole (for STH) and praziquantel (for SCH) in eligible SAC after the last round of MDA supported by TCC in 2021 and the first round of MDA after mainstreaming to state and local government ownership in 2022. Each survey was undertaken within 1 month after MDA. The samples were drawn independently for each district and at each time point. The target MDA coverage for SCH and STH control was 75%.^20^

The sample size for each survey was calculated using the Survey Sample Builder v. 2.10, which creates a multistage cluster sample using population proportionate to estimated size.^21^ The expected coverage was estimated at 70%; we anticipated a 10% nonresponse rate, and we expected 1.9 eligible children per household.^22^ Because MDA coverage tends to be highly clustered, we used a design effect of 4. These parameters yielded a target sample size of 1,435 per district. The sample frame was the complete list of communities provided by the local ministry of health.

Thirty communities were sampled in each district. Approximately 25 households were sampled systematically per community. Schoolchildren may have received MDA according to their school class rather than their age; therefore, we did not exclude children from the coverage survey based on age. All children under age 18 years in selected households were asked to participate after their parent/guardian gave consent. Children also consented/assented to be interviewed. No biological samples were taken. Nonetheless, children aged 5–14 years were the population of interest and are the population identified as “school-aged children” (SAC) for the majority of analyses in this report.

Coverage data responses were collected electronically using Android devices and the ODK-based platform NEMO (https://getnemo.org/). Data were cleaned and analyzed using Stata (StataCorp, College Station, TX). Children were asked both whether they were offered and whether they took mebendazole and praziquantel. Those who were not offered or did not take either drug were asked to select any reasons why not. Given that MDA was school based, children were asked for details about school enrollment, attendance, and type. School attendance was defined as “good” for those attending “always” or “most of the time” and poor for those reporting lower frequencies of attendance. Poor attendance was relatively uncommon, so these children were grouped with unenrolled students for most analyses. School type was initially classified as public, private–faith-based, private–other, boarding, and other. This was dichotomized as public versus other for most analyses. Sociodemographic data were collected at the household level. Wealth quintiles were created for the entire set of households in the study population using the methods adopted by the Demographic Health Surveys.^23^ Household wealth scores were calculated as a weighted sum of various indicator variables, with the weights for each indicator derived from the first component in principal components analysis. Quintiles were applied to each household based on the scores of all individuals in the study population at each survey round. These indicators were collected because SCH and STH are known to affect children from poor backgrounds more often, and we wanted to determine if these children had less access to treatment.^24^^–^^26^

The primary outcomes for STH and SCH were whether the SAC reported taking mebendazole or praziquantel, respectively. In Stata, svy procedures were used for final analyses and estimated CIs to account for the complex sampling design. Weighted coverage estimates were calculated by district among SAC for each drug, then the pre- and post-mainstreaming coverage proportions were compared with an adjusted Wald test.

### Qualitative study.

#### Qualitative study of the mainstreaming process.

Participants were selected purposively according to their role and familiarity with the NTD program. A detailed description of participants is available in Supplemental Tables 1 and 2. Key informant interviews and focus group discussions (FGDs) were conducted by trained facilitators, who were a mixture of Carter Center staff, personnel from the state ministry of health, and consultants. All transcripts and audio recordings were stored in Microsoft SharePoint (Redmond, WA) with protected access.

The discussion topics included strengths and weaknesses of the MDA program and suggestions for mainstreaming, particularly as the study was launched. This feedback was incorporated into the planning and advocacy meetings coordinated by TCC. Interview and discussion guides were adjusted to align with where districts were in the mainstreaming process.

We performed thematic analysis of the interviews and FGDs. A codebook was iteratively developed through reading of transcripts until topic saturation was reached, and the codebook was considered final. Key themes in the data were conceptualized based on topics that emerged from coded segments. Organization, note taking, and thematic analysis of the transcripts were carried out using MaxQDA 2022 software (VERBI GmbH, Berlin, Germany).

## RESULTS

### Mass drug administration coverage surveys.

We interviewed 7,759 children aged 18 years and under across 2,685 households for the pre-mainstreaming survey in 2021. Of these children, 5,441 were aged 5–14 years and were considered SAC (Table 2). The 2021 SAC sample was 49.0% female, and 94.2% of SAC reported good school attendance, defined here as always or most of the time, 0.7% of SAC reported poor attendance, and 5.0% were not enrolled. Of enrolled SAC, 62% went to public schools, 11% went to faith-based private schools, 27% went to other private schools, and <1% went to boarding schools or other. The 2022 post-mainstreaming survey sampled 7,525 children aged 18 years and under, of whom 5,789 were SAC (Table 2); 48.1% of these were female, and 92.1% reported good school attendance, 1.7% reported poor attendance, and 6.2% were not enrolled. Of enrolled SAC, 54% went to public schools, 21% went to faith-based private schools, 25% went to other private schools, and <1% went to boarding schools or other. The gender distribution did not differ significantly between the districts in either survey round (*P =* 0.59 in 2021, *P =* 0.42 in 2022) or between survey rounds in any of the districts (*P =* 0.12 for Ughelli South, *P =* 0.72 for Egor, *P =* 0.65 for Wamba, and *P =* 0.75 for Bassa). Household wealth of the SAC differed between the districts for both surveys (*P <*0.001 in 2021 and 2022), with the residents of Egor, a semi-urban district, tending to fall into the wealthiest quintiles compared with residents of other regions. Wealth quintiles were assigned using scores calculated within each survey, and therefore between-survey statistical comparisons were inappropriate. School attendance also varied significantly by district in both 2021 (*P <*0.001) and 2022 (*P <*0.001). The frequency of good school attendance versus poor attendance/unenrolled was not significantly different between 2021 and 2022 survey rounds overall (*P =* 0.20), though the differences were significant between survey rounds in Ughelli South (*P =* 0.01), Egor (*P =* 0.0001), and Wamba (*P <*0.0001), but not in Bassa (*P =* 0.76). During the pre-mainstreaming survey in 2021, the vast majority of SAC who were “not enrolled” in school were from Bassa (218 of 273, 79.9%); in 2022, unenrolled SAC were relatively common in both Bassa (170 of 358, 47.5%) and Wamba (143 of 358, 39.9%). The numbers remained small in both survey rounds for Ughelli South and Egor (<5% poor attendance/unenrolled). The percentage of SAC enrolled in public schools relative to private/other was significantly higher in the pre-mainstreaming survey (65.1% in 2021 versus 52.2% in 2022, *P =* 0.003), attributable primarily to statistically significant decreases in the percentage of SAC enrolled in public schools between the pre- and post-mainstreaming rounds in Ughelli South (65.1% in 2021 versus 51.1% in 2022, *P =* 0.03) and Wamba (86.7% in 2021 versus 66.9% in 2022, *P =* 0.01). Supplemental Table 1 includes the distribution of SAC by these variables.

**Table 2:** Mass drug administration coverage survey participants before and after mainstreaming, 2021–2022

	Pre-Mainstreaming (2021)			Post-Mainstreaming (2022)		
District	Households Included	SAC Interviewed (5–14 years old)	All Children ≤18 Years Old	Households Included	SAC Interviewed (5–14 years old)	All Children ≤18 Years Old
Bassa	652	1,405	2,063	712	1,293	1,891
Egor	645	1,005	1,513	821	1,547	1,705
Ughelli South	691	1,267	1,840	764	1,507	1,862
Wamba	697	1,764	2,343	668	1,442	2,067
Total	**2,685**	**5,441**	**7,759**	**2,989**	**5,789**	**7,525**

SAC = school-aged children.

Bold values are indicated in total.

### Mebendazole coverage.

**Figure 1.**
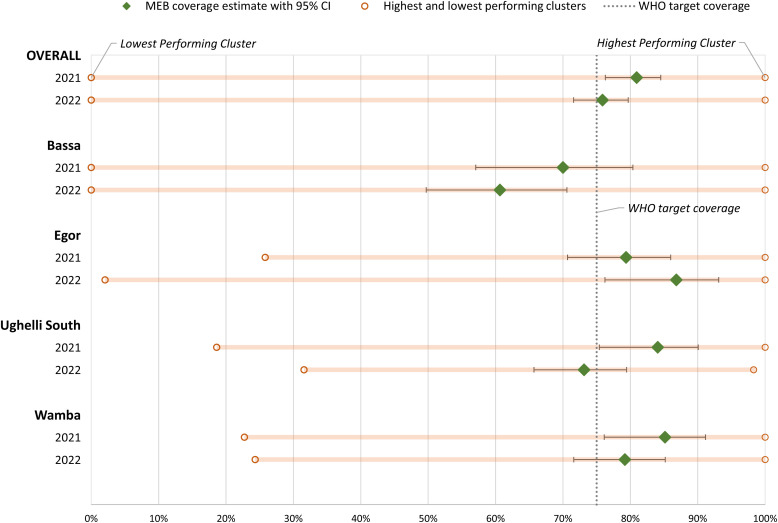
Estimates of mebendazole coverage among school-aged children in four districts of Nigeria before (2021) and after (2022) mainstreaming. MEB = mebendazole.

**Figure 2.**
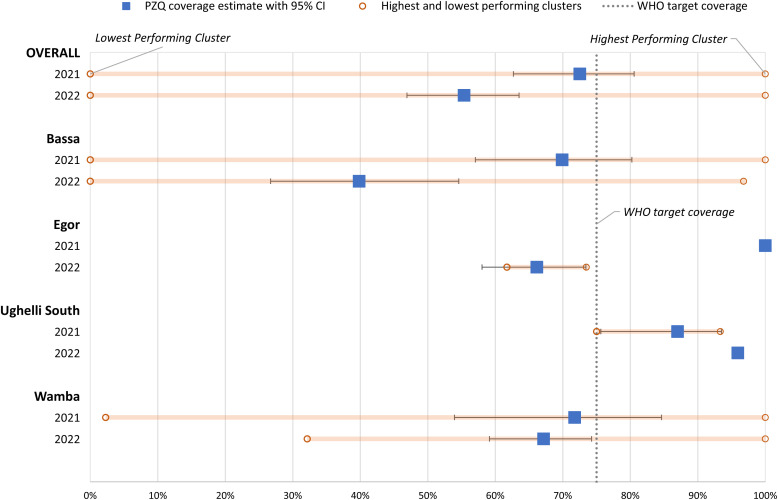
Coverage estimates from household surveys conducted before and after mainstreaming, 2021–2022. PZQ = praziquantel.

Across all four districts, mebendazole (for STH) coverage declined nonsignificantly (*P =* 0.093) from 81% (95% CI: 76–85%) pre-mainstreaming (2021) to 76% (95% CI: 72–77%) post-mainstreaming (2022). Figure 2A shows the estimated coverage by district and survey round for mebendazole among SAC. Mebendazole coverage increased in Egor and declined post-mainstreaming in Bassa, Ughelli South, and Wamba, but the difference between survey rounds was only significant (*P =* 0.03) in Ughelli South (Figure 1, Supplemental Table 3). The coverage estimates did not differ significantly between districts during the pre-mainstreaming round (*P =* 0.14) but differed significantly after mainstreaming (*P =* 0.001), with post-mainstreaming coverage low in Bassa and high in Egor. Coverage was heterogeneous by cluster or village, within districts, as depicted in the wide range from the lowest to highest performing clusters in Figure 1.

Mebendazole coverage did not differ by gender overall or within any district (Supplemental Table 3). The relationship with wealth category was inconsistent. There was no significant relationship between wealth quintile and mebendazole coverage overall or in Wamba or Egor. In Egor, there were no SAC who fell into the two lowest wealth quintiles in either survey. In Bassa and Ughelli South, there was a tendency toward lower coverage with the higher household wealth category, though the trend was significant only in Ughelli South (*P =* 0.01 in 2021; *P <*0.001 in 2022).

The strongest, most consistent predictor of mebendazole coverage was school enrollment/attendance both pre- and post-mainstreaming (Supplemental Table 3). School-aged children who were not enrolled in school or who had poor attendance had consistently low mebendazole coverage in both surveys and across nearly all districts. The only exception was in Egor, but nearly all SAC in Egor reported good school attendance during both surveys (96.2% in 2021 and 99.5% in 2022). Mebendazole coverage also tended to be significantly higher for SAC enrolled in public schools than for those in private/other schools, though the differences were not universally significant. The difference was large and significant pre-mainstreaming but not post-mainstreaming in Wamba (90% versus 57%, *P <*0.01 in 2021; 86% versus 81%, *P =* 0.1 in 2022) and Egor (89% versus 76%, *P <*0.01 in 2021; 90% versus 86%, *P =* 0.26 in 2022); was significant post-mainstreaming but not pre-mainstreaming in Bassa (80% versus 77%, *P =* 0.61 in 2021; 73% versus 36%, *P <*0.01 in 2022); and was significant in both surveys in Ughelli South (91% versus 72%, *P =* 0.001 in 2021; 83% versus 66%, *P =* 0.006 in 2022). Ughelli South, the district where mebendazole coverage significantly decreased between the pre- and post-mainstreaming surveys, also included significantly fewer SAC in the post-mainstreaming survey than in the pre-mainstreaming survey who had good school attendance and who were in public schools. In an exploratory logistic regression, the odds of taking mebendazole in the coverage survey were still lower post-mainstreaming than pre-mainstreaming in Ughelli South after adjustment for school attendance and type, but the decrease between survey rounds was no longer statistically significant (odds ratio = 0.61, *P =* 0.14).

Lack of access, rather than personal/guardian choice or eligibility, was the primary driver of low coverage among SAC both pre- and post-mainstreaming. Only 1.4% of SAC (95% CI: 1–2%) pre-mainstreaming and 1.2% (95% CI: 1–2%) post-mainstreaming said they were offered mebendazole but did not swallow it. School-aged children who did not take mebendazole were asked to select any contributing reasons. Access-related reasons (e.g., not being offered mebendazole, no one coming for MDA, not hearing about MDA, not having MDA offered to their school class/age group, and absence from school when MDA occurred) were most common, being selected by 95% of the SAC who did not take mebendazole in the pre-mainstreaming round and by 93% of SAC who did not take it in the post-mainstreaming round.

### Praziquantel coverage.

Although STH infections are ubiquitous in the study areas, SCH infections are much more focal. Given this, Nigeria targets praziquantel MDA for SCH only to subdistricts, called *wards*, that are classified as SCH endemic. Thus, the coverage survey for praziquantel applied to a limited subset of selected communities that fell into endemic wards and had lower sample sizes/power than the coverage survey of mebendazole for STH. Table 3 indicates the number of communities and participants included in the praziquantel (SCH) coverage surveys. Schistosomiasis is widely distributed in Wamba and Bassa, but there are few relevant communities in Egor District, where only two of 10 wards are endemic for SCH, and Ughelli South District, where only one of 12 wards is endemic for SCH. Between one and three SCH-endemic communities were randomly selected per time point in these two districts.

**Table 3:** Communities and individuals included in the coverage surveys of praziquantel distributions for schistosomiasis

	Pre-Mainstreaming (2021)			Post-Mainstreaming (2022)		
District	Proportion of Villages Targeted with PZQ MDA	All Children ≤18 Years Old in Targeted Villages	SAC (5–14 years old) in Targeted Villages	Proportion of Villages Targeted with PZQ MDA	All Children ≤18 Years Old in Targeted Villages	SAC (5–14 years old) in Targeted Villages
Bassa	30/30	2,159	1,405	30/30	2,257	1,293
Egor	1/30	48	27	2/30	144	130
Ughelli South	3/30	180	123	1/30	55	172
Wamba	17/30	1,388	1,037	15/26	1,410	910
Total	**53/150**	**3,775**	**2,592**	**48/116**	**3,866**	**2,382**

MDA = mass drug administration; PZQ = praziquantel; SAC = school-aged children, 5–14 years old.

Bold values are indicated in total.

Overall, praziquantel coverage among SAC declined significantly (*P =* 0.008) from 73% (95% CI: 63–81%) in 2021 to 55% (95% CI: 47–63%) in 2022 (Figure 2, Supplemental Table 2). Interpreting differences in Egor and Ughelli South was limited given the small numbers of communities that were targeted for praziquantel MDA in these districts, but the coverage appeared higher post-mainstreaming than pre-mainstreaming in Ughelli South and lower in Egor. In Wamba, where the majority of communities were targeted for praziquantel MDA, there was only a slight, nonsignificant decrease (*P =* 0.61) in praziquantel coverage from 72% (54–85%) in 2021 to 67% (59–74%) in 2022. The overall significant decline was primarily driven by the results in Bassa, where all surveyed communities were targeted for praziquantel MDA in both rounds. There was a strongly significant decrease (*P =* 0.002) in coverage from 70% (57–80%) in 2021 to 40% (27–55%) in 2022.

The coverage numbers, particularly post-mainstreaming in 2022, were depressed by praziquantel-specific supply chain problems that led to a complete lack of praziquantel distribution in some communities. In all communities targeted for praziquantel distribution, mebendazole coverage decreased post-mainstreaming, but the difference was smaller and not statistically significant (i.e., 78.5% [95% CI: 70.5–84.8%] took mebendazole in 2021 versus 70.8% [95% CI: 62.4–78.0%] in 2022, *P =* 0.16). In 2021, two of the 30 communities had 0% praziquantel coverage among SAC; in 2022, this number rose to 10 of 30 communities. When the communities with 0% coverage were excluded, praziquantel coverage decreased from 74% (62–83%) in 2021 to 55% (40–70%) in 2022 (*P =* 0.054).

The small numbers of targeted communities and relevant SAC in Egor and Ughelli South limited the ability to analyze and interpret the predictors of coverage, so our assessment concentrates on the districts where SCH was widespread, Wamba and Bassa. All details are included in Supplemental Table 2. The predictors of praziquantel coverage were largely consistent with those of mebendazole coverage. Gender was not an important predictor of praziquantel coverage overall or in Wamba and Bassa (Supplemental Table 2). There was no consistent trend with wealth category and praziquantel coverage. Although there was a slight but statistically significant trend of increasing coverage with wealth category overall post-mainstreaming (*P =* 0.03), there was no clear or statistically significant trend with wealth in any of the specific districts in either survey. Good attendance in school was the strongest and most consistent predictor of higher praziquantel coverage compared with SAC who were not enrolled or who reported poor attendance (both surveys, *P <*0.01). Public schools had higher coverage than private and other types of schools in Bassa, though the differences were not significant, and significantly higher coverage during the pre-mainstreaming survey in Wamba (*P =* 0.002). Post-mainstreaming in Wamba, public schools actually had slightly lower coverage than private and other schools, though this difference was not statistically significant.

Low praziquantel coverage was attributable more to lack of access than to choice or eligibility. In both the pre- and post-mainstreaming rounds, <1% of SAC reported that they were offered praziquantel but chose not to take it. Of the SAC who did not take praziquantel, 98.8% pre-mainstreaming and 95.6% post-mainstreaming indicated that at least one access-related reason contributed to them not taking it. The proportion of SAC who indicated they did not take praziquantel because they did not hear about the MDA or because no one came to administer it increased significantly from 4.4% pre-mainstreaming to 28.8% post-mainstreaming (*P <*0.001), though this variable is difficult to interpret as more than a third of SAC pre-mainstreaming and more than half of SAC post-mainstreaming said they did not know or could not remember why they did not get praziquantel.

### Qualitative study.

We conducted KIIs and FGDs in three phases during the study. Participants were key staff within the health and education systems as well as focus groups with community members familiar with the MDA program. The distribution of respondents is shown in Table 4. Focus group discussions ranged in size from three to 11 participants, generally divided by gender.

**Table 4:** Participants in qualitative interviews and focus groups

Phase	Pre-Mainstreaming (July 2021)		During Mainstreaming (April/May 2022)		After Mainstreaming (July/August 2022)	
State	KIIs	FGDs	KIIs	FGDs	KIIs	FGDs
Edo	22	9	19	8	22	2
Delta	25	3	13	12	9	5
Nasarawa	14	5	10	7	10	6
Plateau	10	3	11	8	8	6
Total	71	20	53	35	49	19

KII = key informant interview; FGD = focus group discussion. Note: Some participants were interviewed multiple times throughout the study.

Key themes in the data were the importance of funding, drugs and other resources, training of NTD program personnel, community and stakeholder advocacy and overall program planning, communication, and monitoring. Participants shared suggestions for successful program continuation. In general, respondents were opposed to mainstreaming and government ownership. Participants also shared their expectations and desires for the mainstreaming process prior to transitioning, followed by their perception of the process during and after.

#### Before mainstreaming.

The STH and SCH MDA programs were overwhelmingly popular among interviewees and focus group participants. Respondents spoke of the health benefits that these medications brought to children in their communities. One participant celebrated that “my people do not suffer from some of the NTDs anymore, so that is a personal gain for me. TCC activities led to the eradication of guinea worm, and at the moment, transmission of LF has been interrupted, and still the fight is on against other NTDs” (NASARAWA 9-Education, State). Informants expressed gratitude to TCC for the training that prepared them to administer drugs properly and educate parents about the importance of MDA. Participants often overstated the role of TCC in MDA, at times attributing all logistics and management aspects to the NGO rather than the health system.

The respondents in the qualitative portion of the study categorically felt negatively about mainstreaming, hoping instead that TCC would maintain its involvement at some level or slowly phase out involvement. “The Carter Center should not hand over and hands off entirely because no one can drive a vision better than the visioner” (EDO 2-Education, LGA). Participants’ pessimism about mainstreaming centered on financing, stakeholder coordination, and grassroots advocacy, all of which were seen as lacking within the government’s MDA program despite its popularity. According to one participant:

*“I think if TCC withdraws from the NTD program, that will be the end of the fight against NTDs because I have seen how government is finding difficulty supporting some aspects of the program. I am of the opinion that TCC should not hand-off the program completely, but should be involved in some of the activities such as training, treatments, etc., until the relevant ministries and agencies are ready to take full charge of the program. I am almost certain if TCC pulls out, the NTD program will only be on paper, and it will not be effective as it used to be. I have seen a couple of programs ended abruptly due to lack of funds…” (NASARAWA 3-Health, LGA)*.

Key informants pleaded for TCC to gradually transition full NTD program leadership to the government after sufficient preparation and for TCC to continue to supervise and support the program for many years into the future. “I will prefer a transfer that is done in bits, so that we see how the people can handle the different aspects. So, like once you let go of an aspect, we see how it goes, you let go of another aspect, we see how it goes, and so on” (EDO 21-Health, State). Financial support from TCC was frequently mentioned and was viewed as necessary for drug acquisition, distribution, and administration. Respondents also mentioned the stipends given to NTD staff for their time spent in training, transportation reimbursement, and labor during drug administration. Such support was recognized as a great facilitator and motivator for program participation: “If stipends for refreshment are not made available or a lesser amount is paid, the CDDs [community directed distributors] and teachers will not be as committed as they used to be” (NASARAWA 1-Education, LGA). Participants regularly implied that personnel would not focus on MDA if financing were withdrawn. Participants encouraged TCC to advocate heavily with government officials to sustain financial support; ministries were often viewed as unmotivated and uninvolved. In Edo, one informant shared the following:

*“Governments of the day are not playing any role in improving health in the society…when this handover is done, things will definitely be different…the NTD program might be affected negatively after the handover…. Government may not release adequate fund on time, the attitude of the worker may change because they are not being paid their due, corruption and embezzlement will then come into play” (EDO 8-Government, LGA)*.

Corruption, poor job performance, and an “‘I don’t care’ attitude seen in government workers” (EDO 7-Education, LGA) were expected to lead to drug shortages and decrease financial benefits for workers in the MDA program.

Funding was not the only source of concern for participants. They viewed TCC as the glue among the various components in program operations, providing support for planning, coordination, and monitoring and evaluation in addition to MDA activities. “They have acted as intermediary anyway from what I see, their role is important kind of, to bring both parties together, it’s like having a glue that brings people together… (EDO 2:1-Education, LGA).” Key informants called on TCC to prepare the government for the NTD program, ideally over a long, gradual period. They recommended that TCC provide guidelines on how to maintain the current structure, clearly spelling out the roles of those in the legislative and executive arms of government, local government agencies, and other stakeholders. Many proposed that TCC assist in drafting a memorandum of understanding to push for accountability among parties. Informants wished for preparation to also include budgeting, technical management capacity building, training, and program planning: “We do not have the monitoring and evaluation system [or] structure needed for the program to succeed” (NASARAWA 9-Health, State).

#### During the mainstreaming transition.

During the mainstreaming process, participants in interviews and FGDs continued to applaud the success of MDA programs in improving the health of their states’ children, but respondents talked more frequently about the program’s shortcomings than in the first round of data collection. They also observed differences compared with previous rounds. One participant stated, “The advocacy has not been so impressive like the previous [round of MDA] where [The Carter Center] was fully doing everything” (NASARAWA 2:3-Education, State). Participants noticed the lack of training going into the post-mainstreaming MDA and recognized this as a problem moving forward. “Trained personnel, sometimes they are few. They lack them due to retirement from service. Most of the trained personnel that are involved, if they go we don’t have trained personnel on [the] ground. They need another batch of training to fill in that gap” (PLATEAU 2:4-Education, State).

Expectations for government-run MDA remained negative for the majority of respondents. Broadly, the government was thought of as incapable of running the program in the same fashion. One person shared a common sentiment: “It will never go smoothly, because there are so many services the government [is] supposed to render to its citizens, but they don’t, therefore TCC should continue with the program” (Nasarawa 2 FGD 1-Women). Most beneficiaries agreed that “nothing excites me about the mainstreaming,” with one person predicting that “we are going back to square one, there will be more suffering and the diseases will come back” (NASARAWA 2 FGD 1-Women).

Government funding of the program remained the primary source of concern, and participants were worried about its effects on drug acquisition and distribution, the payment of program staff, and the organization of trainings and other current program components. A few participants cited lack of payment to government employees, with one explaining that “the government workers are suffering from non-payment of salaries, so you can imagine if they decide to take over. The government will fail in this course” (PLATEAU 2 FGD 1-Female teachers). Participants were unsure how drugs would be supplied and were doubtful that funds or personnel would be released for training.

In the lead-up to the second round of MDA, participants gave many suggestions and pleas for ways to mitigate the negative impacts they foresaw from government ownership. Many of these included temporary or permanent continuation of TCC ownership, as one suggests “they should not hand over the program completely to the government but continue to be there running the program and then withdraw gradually. Also, the government should be engaged in counterpart funding. In fact, there should be no withdrawal—TCC should continue running the program (DELTA 2 FGD 10-Men). Respondents also asked that additional training and advocacy be done before withdrawal.

#### After mainstreaming.

Most participants were displeased with how the MDA was carried out under government ownership. They observed changes in the breadth of drug distribution, training of staff, and payments to staff. “There is a lot to be afraid of. As you can see this year, it is very visible that the government cannot handle this program like the way [The] Carter Center has handled it. These drugs did not get to so many places and if that continues, these diseases will come back as a result of this mainstreaming,” said one Plateau resident (PLATEAU 3 FGD 1-Female community drug distributors). Another participant stated, “Seriously, some of the children did not get the drugs. Even the children in the schools were complaining they did not get the drugs while others were able to get. They said the drugs were not shared the way it is used to” (NASARAWA 3 FGD 3-Women). Participants also described a decrease in morale among communities and program staff, with one stating, “Well, the thing that changed negatively was the enthusiasm has dropped, motivation has dropped so the implementation time was slow” (NASARAWA 3:4-Health, State). Respondents perceived that only the minimum number of activities occurred, and they did not expect the situation to improve on its own.

*“There must be challenges when you are starting something for the first time. Both health staff and teachers are used to being motivated with allowances and if these allowances are withdrawn, there will be problems. Like in the case of supervision, it will not be there. The training wasn’t done this year, supervision itself wasn’t done either and I think it was quite difficult for teachers to collect their medicine to distribute as it used to be. In Wamba LGA, we added more facilities so that the collection centres will be close to the teachers and yet, some of the teachers were still complaining about not having transport to go to the facilities to collect their medicines. These were some of the challenges. Another challenge is the fact that some of the teachers that were trained last year were no longer working so, it was difficult for the new teachers to cope” (NASARAWA 3:3-Health, State)*.

Communities noticed changes in the post-mainstreaming round of MDA, agreeing that school authorities, the Parent Teachers Association (PTA), and State Universal Basic Education Board (SUBEB), depending on the local government, took over many of the MDA tasks. This included both drug transportation and distribution as well as funding. One person reported that “The Carter Center used to employ the use of CDDs to carry out [drug collection] but this time around, it was the teachers that were called to go pick up the drugs in some health activities to distribute” (PLATEAU 3 FGD 2-Female teachers). Another described how the PTA began to fund the activities: “You see, we have this allowance or dues that those bodies used to pay in schools as [a] PTA or SBMC [school-based management committee] levy, it’s a levy. These levy or contributions [are] not meant for this program, but we agreed to use a little out of it for the distribution of these drugs” (PLATEAU 3:2-Education, State).

These collaborations and shifts in responsibility were viewed skeptically. According to one participant:

*“Their collaboration in some areas are very weak because if the collaboration is strong enough we will not be seeing the level of this failure we are witnessing today. The ministry of education and SUBEB failed to understand that without good health, there can be no education, they lack respect for one another, and one body cannot work independently without the other so they need to collaborate so that we will achieve success. Parent-Teacher Association (PTA) and the teachers are in the schools, there should be understanding between them. Going forward, proper planning and adequate information should be given to the PTA about what is expected of them so that our children can take these drugs” (PLATEAU 3 FGD 3-Male community drug distributors)*.

Many were dubious that any gains could be maintained in the face of mainstreaming, noting systemic challenges and overwhelmed staff. During an FGD in Plateau, one participant summarized as follows:

*“Honestly, if you will check or look at the differences of last MDA and the current MDA, now that The Carter Center has withdrawn and handed over the program to the government, we have serious challenges, when we have not even gotten anywhere. Now that we have these problems, do you think if this program has been left for the government we will have progress? Even the teachers don’t get their pay for teaching time, school are always on strike, while on strike do you think these teachers will follow you home with these drugs? Please, Carter Center should look into these issues” (PLATEAU 3 FGD 3-Male community drug distributors)*.

Although most community members were dissatisfied with the decision to mainstream and with the outcomes of the MDA program, some had begun to accept that this was the way of the future. According to one person:

*“Well, I look at the transition from The Carter Center to the government in this way, if you are hungry and food is brought before you, you don’t expect the person that brought the food to hold your hands to fetch the food and put in your mouth. You need to feed yourself to get satisfied. Now, we have these diseases within our communities and Carter Center must not do everything for us. They have done enough and if they feel they want to now hand over, we do not have the strength to say no, rather, we should embrace it and know how we can take care of ourselves in this aspect” (NASARAWA 3:3-Health, State)*.

Another said:

*“For me, I think there should be no going back. [I]nitially I was hoping and praying we would push the support so that we get stronger before but you know like you are teaching a child to walk, the child will stumble, the child will fall but the child will pick himself up and walk again. For me I think the transition should continue. We may go back and forth, we may lose some momentum but we will pick up as we go because if The Carter Center comes back again that means [it] is like a child that is learning to walk because he falls down and you said no sit down it doesn’t work like that. You know you keep encouraging the child to walk again. For me I think we should proceed with the transition probably repeat one or two circles and get stronger in Wamba LGA then we can scale up” (NASARAWA 3:4-Health, State)*.

Many other people shared ideas for improving the program under its new ownership as well. When considering the need for more funding, one informant suggested the following:

*“The challenges for me I think, what I would have loved to see is that if we have anticipated the effect of the stipends on these CDDs maybe we would have made provision for the stipends but again as a public health person I think I am looking at the scenario where we entrench a sustainable system. So maybe what I would have advocated is that we innovate a different rewarding system, so that [it] is not dependent on stipends but again the transportation is a challenge so we either transport them or provide the transport stipends. Maybe going forward, [it] is for us to know how much is to be spent in providing that logistics and then see if we can provide it” (DELTA 3:4-Education, LGA)*.

Although most participants were dubious of others’—and even their own—commitment to the program in the absence of NGO funding, many recognized that they would be responsible for carrying it themselves and did not balk from this effort. They surmised it would take passionate commitment despite the challenges presented by mainstreaming. In one FGD, respondents committed to working tirelessly, without pay, and actively engaging with religious leaders to mobilize resources. “I will sacrifice my energy, time, and resources. This is because I have my community at heart” and “I will give myself and time in doing this work, this is because these children are our tomorrow leaders, so we have to protect and guard them” (NASARAWA 3 FGD 6-Female health workers).

Integrated campaigns, that is, co-delivering SCH/STH medicines with other interventions, received mixed feedback. Some people expressed confidence that campaign integration would make drug distribution, training, and monitoring more efficient and cost-effective, whereas others did not find the premise to be sound, stating that “in any integrated program, the personnel need to be very active because handling two or three activities is not easy. Integration tends to broaden the knowledge of such personnel and again it is causing more headache. In our schools a teacher who is supposed to handle class work is now administering drugs. Reporting may come with some errors in the data” (PLATEAU 3 FGD 4-Male teachers). It was widely agreed that more training and manpower would be needed for mainstreaming via campaign integration to be successful, and that logistics were not straightforward:

*“In the LGA there are a lot of programs, like Maternal and Child Health week, which takes place twice in a year. Malaria community work, which is done by the PMI, Presidential Malaria Initiative. Seasonal Malaria Chemoprevention, SMC, is also being sponsored by an NGO. So, if we are to integrate SCH/STH treatment into any of these programs, SMC is just for two years that means at the end of two years it will stop. So, integrating with any other program for sustainability, will not go well with us. Because most of these programs stop. The Carter Center has done a good job from the beginning why should we go and spoil what they have been doing. So, integrating this program, I think will not work well” (NASARAWA & PLATEAU 3 FGD 1-Health)*.

## DISCUSSION

Our study demonstrated that MDA coverage for STH did not decline significantly after the withdrawal of NGO support, also known as mainstreaming, despite intense pessimism observed during qualitative interviews with key informants. The coverage results were less promising for SCH coverage, which did decline significantly post-mainstreaming, though results were confounded by a shortage of praziquantel in 2022. For both medications, there were few reported refusals, with most participants citing a lack of access as the reason they did not take MDA. There are many factors supporting these results.

First, this is a mature program evaluated early in the mainstreaming experience after only a single round of MDA. The results could be substantially different after more time passes and staff depart. Indeed, our qualitative study discovered that staff had benefitted from many years of repeated training and attention from TCC. This provided a buffer period between full NGO support to a truly country-run program. Repeating surveys in a few years after new processes have been established and after staff turnover would give a truer picture of mainstreaming and the outcomes of a fully country-run program.

Second, TCC maintained support for drug transport so that medicines were available to be distributed to schools. Funding and logistical support are critical for the MDA to even begin, and challenges upstream—as evidenced by the significant decrease in praziquantel coverage—are beyond the scope of local schools and officials or an NGO to solve. A previous study showed that when TCC support was withdrawn from a community-based onchocerciasis MDA program in Nigeria, treatments declined and medicine accumulated in storage and was not distributed.^27^ Other approaches to mainstreaming and country ownership in Nigeria have celebrated a true national supply chain,^28^ which was not the approach used in this study. Mechanisms for the transparent, timely, and equitable distribution of medicines must be supported by all parties, and a plan to taper off support with sufficient training elements is preferred over an abrupt withdrawal of all external support. A cost-effective compromise between primary support and complete mainstreaming could be for partners to host less frequent trainings or to focus primarily on training of new staff.

The qualitative components of our study warned of poor outcomes after mainstreaming. These pessimistic predictions were by and large not observed in the coverage data, but the true effects may become apparent in subsequent rounds. Significant reductions in external support elsewhere in Nigeria have shown negative impacts on the quality and breadth of HIV/AIDS services.^29^ Although TCC’s withdrawal of support was not gradual like participants wanted, it was thoroughly discussed, and program staff and diverse stakeholders were deeply involved in discussions and planning. Comprehensive planning with clear articulation of roles and responsibilities should be encouraged.^30^ We must also acknowledge that many of the interviewees have received benefits, financial and otherwise, from engaging with TCC over the years, which may have intensified their pessimism about mainstreaming. Our facilitators represented both TCC and the Ministry of Health, which may have influenced their responses in a variety of directions.

Sustaining achievements, let alone moving toward elimination, requires collaboration across sectors, multipronged interventions, and regular treatment.^31^ Grassroots engagement can maintain community involvement and awareness of MDA, which is critical to keeping treatment coverage high.^32^ Community-based actors are well positioned to educate families and coordinate treatment with synergistic effects,^33^ but these structures will crumble if support from the broader health system does not exist to ensure expertise and availability of medicines.^34^ By directly addressing the structures and roles responsible for reaching SCH/STH control goals, comprehensive mainstreaming efforts could improve the engagement of all participants.^35^ Study participants continually asked TCC to serve as the “connector” among communities and the various official bodies involved in MDA. Such coordination would need to address the behavior, infrastructure, and planning angles needed to sustain positive health outcomes,^36^ which does not align well with a light-touch philosophy of mainstreaming. Indeed, mainstreaming may need to begin not as a withdrawal of external support but as a redirection of technical assistance. Although the vertical campaigns of NTD programs have proven effective, they are often not designed to improve primary healthcare systems.^37^

Domestic financing, a pervasive theme in the qualitative results, is a critical component of program ownership^38^^,^^39^; strategies to reduce costs include integrated impact surveys, reduced frequency of MDA (if prevalence is sufficiently low), incorporating deworming into a package of school health interventions delivered at specific school milestones, and supporting improvements in sanitation and hygiene.^40^^–^^42^ Many programs have used integration of NTD activities to share funding and unlock domestic financing, although the amounts have generally been insufficient to sustain activities independently.^43^ Countries have largely relied on donors and partners to financially support NTD programs.^44^^,^^45^ The progressive elimination of onchocerciasis, LF, and trachoma shifts the internal and external funding picture considerably for endemic countries, many of which have integrated training for all NTDs based on external funding for these (now disappearing) diseases.^46^

Strategies for program integration were described by the participants in the qualitative study with mixed enthusiasm. Participants were fully aware that their programs were largely carried out via external funding and that there was minimal domestic appetite to turn away from such support.^47^ They recognized that true mainstreaming would require significant structural changes within the health system,^48^ a challenging proposition in any environment, let alone one as complicated as Nigeria.^49^ Nonetheless, the participants in this study were unified in their commitment to the health of children and their ongoing protection from SCH and STH, confirming that there is energy for creative solutions to the challenge of mainstreaming.

## Supplemental Materials

10.4269/ajtmh.23-0600 Supplemental Materials
