# Supplementary material for: A Mixed-Methods Evaluation of Mainstreaming Mass Drug Administration for Schistosomiasis and Soil-Transmitted Helminthiasis in Four Districts of Nigeria
Source: Am J Trop Med Hyg. 2024 Apr 9;111(3 Suppl):69–80. doi: 10.4269/ajtmh.23-0600 (PMC11376119; doi:10.4269/ajtmh.23-0600)
Supplement: Supplemental Materials [file tpmd230600.SD1.pdf]

Supplemental Table S1

Selection Criteria for Participation in Qualitative Study

Group	Participants
Key Informant Interviews	Participants were selected based on their position in the program as government officials in ministry of health, Primary Health Care (PHC), Education, and Universal Basic Education Boards. They had direct roles and responsibilities in the decision making, approval processes, fund releases, planning, implementation, monitoring and supervision, training of teachers and community drug distributors (CDDs), and reporting. These officers were selected at State and LGA levels. At state level, permanent secretaries, directors, NTD program coordinators and team members were included. At the Ministry of Education, similar cadres were selected. At planning and economic/budget departments, Finance and information ministries, we interviewed consenting directors. At the LGA level, we interviewed the LGA heads of administration, Directors of PHC, NTD Program officers as well as their team members. In the LGA, we interviewed education secretaries and Chief inspectors of education. At the ministry of Education, we interviewed directors of schools or any desk officer in charge school health services.
Focus Group Discussions	Discussions were organized for the distributors - teachers, CDDs, parents-teachers associations, community leaders (male and female), and frontline health workers who distribute or train CDDs or involved in drug logistics.

Supplemental Table S2

Distribution of Key Informant Interviews by Participants' Area of Responsibility

	**Pre-Mainstreaming**			**During Mainstreaming**			**Post-Mainstreaming**		
	Education	*Government*	*Health*	*Education*	*Government*	*Health*	*Education*	*Government*	*Health*
**Delta**									
State	5	2	8	2	1	10	2	0	2
LGA/Community	5	1	4	0	0	0	2	1	2
**Edo**									
State	10	0	7	1	0	9	4	0	6
LGA/Community	2	1	2	2	2	5	3	0	9
**Nasarawa**									
State	2	0	2	0	0	0	0	0	6
LGA/Community	5	1	4	3	4	3	2	0	2
**Plateau**									
State	3	0	5	5	1	5	3	0	5
LGA/Community	0	0	2	0	0	0	0	0	0
**TOTAL**	**32**	**5**	**34**	**13**	**8**	**32**	**16**	**1**	**32**

In total, 173 Key Informant Interviews were conducted across the study.

Table S3. Mebendazole Coverage Among School-Aged Children by Characteristic and District, Pre- and Post-Mainstreaming

	Pre-Mainstreaming (2021)				Post-Mainstreaming (2022)				
	SAC (5-14 years) (n)	*Took MEB during MDA (n)*	*Weighted coverage estimate (95% CI)*	*p-value[1]*	*SAC (5-14 years) (n)*	*Took MEB during MDA (n)*	*Weighted coverage estimate (95% CI)*	*p-value[1]*	*Pre- vs. Post- p-value[2]*
District: Bassa									
Bassa Total	1,405	982	69.9% (57.0 – 80.2%)		1,293	784	60.6% (49.9 – 70.5%)		0.25
**Gender**									
Male	723	514	71.1% (58.0 – 81.4%)		673	416	61.8% (50.6 – 71.9%)		
Female	682	468	68.6% (55.5 – 79.3%)	0.26	620	368	59.4% (48.4 – 69.4%)	0.31	
**Wealth Quintile[3]**									
Lowest 20%	359	272	75.8% (62.2 – 85.6%)		544	356	65.4% (53.1 – 76.0%)		
>20-40%	499	338	67.7% (46.7 – 83.4%)		368	227	61.7% (47.3 – 74.3%)		
>40-60%	280	207	73.9% (54.9 – 86.9%)		222	134	60.4% (41.7 – 76.5%)		
>60-80%	176	113	64.2% (48.7 – 77.2%)		112	51	45.5% (15.7 – 78.9%)		
Top 20%	84	47	56.0% (42.1 – 68.9%)	0.29	28	7	25.0% (5.9 – 63.9%)	0.23	
**School Attendance[4]**									
Unenrolled/poor	221	48	21.7% (9.0 – 43.8%)		226	98	43.4% (20.5 – 69.5%)		
Good attendance	1,177	930	79.0% (68.4 – 86.7%)	**<0.01**	1,062	684	64.4% (53.2 – 74.2%)	0.13	
**School Type**									
Private, boarding, other	378	290	76.7% (63.0 – 86.4%)		236	84	35.6% (19.0 – 56.6%)		
Public	796	636	79.9% (67.6 – 88.3%)	0.61	824	602	73.1% (64.6 – 80.1%)	**<0.01**	
District: Egor									
Egor Total	1,005	796	79.2% (70.6 – 85.8%)		1,547	1,343	86.8% (76.4 – 93.0%)		0.18
**Gender**									
Male	510	400	78.4% (69.3 – 85.4%)		774	671	86.7% (76.0 – 93.0%)		

	Pre-Mainstreaming (2021)				Post-Mainstreaming (2022)				
	SAC (5-14 years) (n)	*Took MEB during MDA (n)*	*Weighted coverage estimate (95% CI)*	*p-value[1]*	*SAC (5-14 years) (n)*	*Took MEB during MDA (n)*	*Weighted coverage estimate (95% CI)*	*p-value[1]*	*Pre- vs. Post- p-value[2]*
Female	495	396	80.0% (71.1 – 86.7%)	0.53	773	672	86.9% (76.6 – 93.1%)	0.83	
**Wealth Quintile[3]**									
Lowest 20%	0	--	--		0	--	--		
>20-40%	0	--	--		0	--	--		
>40-60%	15	14	93.3% (52.3 – 99.4%)		68	55	80.9% (54.6 – 93.7%)		
>60-80%	232	194	83.6% (69.1 – 92.1%)		464	433	93.3% (83.8 – 97.4%)		
Top 20%	758	588	77.6% (68.2 – 84.8%)	0.27	1,015	855	84.2% (71.8 – 91.8%)	0.27	
**School Attendance[4]**									
Unenrolled/poor	38	34	89.5% (74.9 – 96.0%)		8	8	100% (n/a)		
Good attendance	963	760	78.9% (70.1 – 85.7%)	0.10	1,538	1,335	86.8% (76.4 – 93.1%)	n/a	
**School Type**									
Private, boarding, other	714	539	75.5% (66.4 – 82.8%)		1,071	917	85.6% (73.1 – 92.9%)		
Public	254	225	88.6% (80.8 – 93.5%)	**<0.01**	438	393	89.7% (82.1 – 94.3%)	0.26	
District: Ughelli South									
Ughelli South Total	1,267	1,065	84.1% (75.5 – 90.0%)		1,507	1,102	73.1% (65.8 – 79.4%)		**0.03**
**Gender**									
Male	622	524	84.2% (76.7 – 89.7%)		796	572	72.2% (64.3 – 78.9%)		
Female	645	541	83.9% (73.9 – 90.5%)	0.85	711	530	74.2% (66.8 – 80.4%)	0.34	
**Wealth Quintile[3]**									
Lowest 20%	384	343	89.3% (83.2 – 93.4%)		210	184	86.0% (73.7 – 93.1%)		
>20-40%	241	224	92.9% (86.4 – 96.5%)		248	206	84.1% (76.2 – 89.7%)		
>40-60%	302	257	85.1% (72.2 – 92.6%)		417	303	72.5% (63.2 – 80.2%)		

	Pre-Mainstreaming (2021)				Post-Mainstreaming (2022)				
	SAC (5-14 years) (n)	*Took MEB during MDA (n)*	*Weighted coverage estimate (95% CI)*	*p-value[1]*	*SAC (5-14 years) (n)*	*Took MEB during MDA (n)*	*Weighted coverage estimate (95% CI)*	*p-value[1]*	*Pre- vs. Post- p-value[2]*
>60-80%	212	146	68.9% (49.0 – 83.6%)		369	252	68.7% (57.5 – 78.0%)		
Top 20%	128	95	74.2% (58.7 – 85.4%)	**0.01**	245	140	57.5% (46.2 – 68.0%)	**<0.01**	
**School Attendance[4]**									
Unenrolled/poor	20	12	60.0% (40.4 – 76.8%)		56	18	30.2% (16.6 – 48.5%)		
Good attendance	1,237	1,045	84.5% (75.7 – 90.5%)	**0.01**	1,430	1,070	74.7% (67.2 – 81.0%)	**<0.01**	
**School Type**									
Private, boarding, other	436	312	71.6% (57.7 – 82.2%)		696	460	65.6% (54.5 – 75.2%)		
Public	814	743	91.3% (84.6 – 95.2%)	**0.001**	710	586	82.8% (74.5 – 88.8%)	**0.006**	
District: Wamba									
Wamba Total	1,764	1,502	85.1% (76.2 – 91.1%)		1,442	1,142	79.2% (71.7 – 85.1%)		0.24
**Gender**									
Male	920	781	84.9% (75.2 – 91.3%)		763	602	78.9% (71.1 – 85.0%)		
Female	844	721	85.4% (76.9 – 91.2%)	0.75	679	540	79.5% (71.4 – 85.8%)	0.80	
**Wealth Quintile[3]**									
Lowest 20%	232	183	78.9% (60.5 – 90.1%)		257	196	76.3% (67.0 – 83.5%)		
>20-40%	339	293	86.4% (76.5 – 92.6%)		373	282	75.6% (61.9 – 85.5%)		
>40-60%	481	429	89.2% (79.6 – 94.6%)		419	329	78.5% (67.7 – 86.4%)		
>60-80%	512	433	84.6% (71.4 -92.3%)		297	256	86.2% (73.5 – 93.4%)		
Top 20%	199	163	81.9% (56.5 – 94.0%)	0.90	84	71	84.5% (64.1 – 94.4%)	0.19	
**School Attendance[4]**									
Unenrolled/poor	34	16	47.1% (28.1 – 67.0%)		166	64	38.6% (23.3 – 56.4%)		
Good attendance	1,717	1,476	86.0% (77.0 – 91.8%)	**<0.01**	1,269	1,075	84.7% (78.9 – 89.2%)	**<0.01**	

	Pre-Mainstreaming (2021)				Post-Mainstreaming (2022)				
	SAC (5-14 years) (n)	*Took MEB during MDA (n)*	*Weighted coverage estimate (95% CI)*	*p-value[1]*	*SAC (5-14 years) (n)*	*Took MEB during MDA (n)*	*Weighted coverage estimate (95% CI)*	*p-value[1]*	*Pre- vs. Post- p-value[2]*
**School Type**									
Private, boarding, other	228	129	56.6% (44.9 – 67.6%)		422	341	80.8% (75.3 – 85.3%)		
Public	1,489	1,346	90.4% (83.0 – 94.8%)	**<0.01**	851	731	85.9% (78.8 – 90.9%)	0.10	
Total for all districts									
Total	5,441	4,345	80.9% (76.3 – 84.9%)		5,789	4,371	75.9% (71.6 – 79.7%)		0.09
**Gender**									
Male	2,775	2,219	81.0% (76.3 – 84.9%)		3,006	2,261	75.4% (70.9 – 79.4%)		
Female	2,666	2,126	80.9% (76.0 – 85.0%)	0.94	2,783	2,110	76.3% (71.9 – 80.2%)	0.42	
**Wealth Quintile[3]**									
Lowest 20%	975	798	83.8% (77.8 – 88.5%)		1,011	736	75.4% (67.7 – 81.8%)		
>20-40%	1,079	855	82.2% (72.4 – 89.1%)		989	715	75.5% (68.5 – 81.4%)		
>40-60%	1,078	907	84.8% (77.8 – 90.0%)		1,126	821	73.3% (66.8 – 78.8%)		
>60-80%	1,132	886	77.8% (69.3 – 84.5%)		1,242	992	78.5% (70.8 – 84.6%)		
Top 20%	1,169	893	76.6% (69.4 – 82.6%)	0.12	1,372	1,073	76.1% (66.8 – 83.5%)	0.70	
**School Attendance[4]**									
Unenrolled/poor	313	110	39.1% (24.4 – 56.1%)		456	188	39.8% (27.7 – 53.3%)		
Good attendance	5,094	4,211	83.0% (78.6 – 86.7%)	**<0.01**	5,299	4,164	78.5% (74.2 – 82.3%)	**<0.01**	
**School Type**									
Private, boarding, other	1,756	1,270	72.1% (65.7 – 77.7%)		2,425	1,802	73.4% (66.7 – 79.3%)		
Public	3,353	2,950	88.8% (84.7 – 91.9%)	**<0.01**	2,823	2,312	82.7% (78.5 – 86.2%)	**0.008**	

	Pre-Mainstreaming (2021)				Post-Mainstreaming (2022)				
	SAC (5-14 years) (n)	*Took MEB during MDA (n)*	*Weighted coverage estimate (95% CI)*	*p-value[1]*	*SAC (5-14 years) (n)*	*Took MEB during MDA (n)*	*Weighted coverage estimate (95% CI)*	*p-value[1]*	*Pre- vs. Post- p-value[2]*

Abbreviations: MDA = Mass drug administration; MEB = Mebendazole; n/a = Not applicable; SAC = School-aged children, 5-14 years
Bolded p-values are those that are <0.05.

1. p-value testing the difference between proportions of coverage by category of gender, wealth quintile, or school attendance within a single round of the survey.
2. p-value testing the difference between proportions in the pre- and post-mainstreaming coverage estimates for the sub-population of interest.
3. Wealth index variables were calculated for each household based on the methods adopted by the Demographic Health Survey (DHS) See Methods in text for details. Quintiles were defined separately for the pre-mainstreaming population and the post-mainstreaming population. The household wealth information was missing for 8 SAC in the pre-mainstreaming round, of whom 6 took MEB, and for 49 children in the post-mainstreaming round, of whom 34 took MEB. The p-value comes from the test of wealth category as the sole predictor in a logistic regression incorporating survey weights, in order to test for a significant trend with increasing wealth quintile.
4. Attendance was classified as "Good" if the student reported attending school 'always' or 'most of the time' and as "Poor" if the student reported attending school "Part-time", "Sometimes", "Rarely", or "Never". The categories of 'Not enrolled' and 'Poor attendance' were collapsed for analysis due to small numbers within districts. There were 34 SAC missing this variable in the pre-mainstreaming round, of whom 24 took MEB, and 34 SAC missing this variable in the post-mainstreaming round, of whom 19 took MEB.

Table S4. Praziquantel Coverage Among School-Aged Children by Characteristic and District in Selected Villages Targeted for Schistosomiasis MDA, Pre- and Post-Mainstreaming

	**Pre-Mainstreaming (2021)**				**Post-Mainstreaming (2022)**				
	SAC (5-14 years) (n)	*Took PZQ during MDA (n)*	*Weighted coverage estimate (95% CI)*	*p-value[1]*	*SAC (5-14 years) (n)*	*Took PZQ during MDA (n)*	*Weighted coverage estimate (95% CI)*	*p-value[1]*	*Pre- vs. Post-p-value[2]*
				District: Bassa					
Bassa Total	1,405	982	69.9% (56.9 – 80.3%)		1,293	515	39.8% (26.6 – 54.7%)		**0.002**
**Gender**									
Male	723	514	71.1% (57.9 – 81.5%)		673	276	41.0% (27.0 – 56.6%)		
Female	682	468	68.6% (55.4 – 79.4%)	0.26	620	239	38.5% (25.7 – 53.3%)	0.44	
**Wealth Quintile[3]**									
Lowest 20%	359	272	75.8% (62.1 – 85.6%)		544	174	32.0% (15.1 – 55.4%)		
>20-40%	499	338	67.7% (46.6 – 83.5%)		368	169	45.9% (29.1 – 63.7%)		
>40-60%	280	207	73.9% (54.7 – 86.9%)		222	108	48.6% (30.6 – 67.1%)		
>60-80%	176	112	63.6% (47.6 – 77.1%)		112	53	47.3% (16.7 – 80.1%)		
Top 20%	84	48	57.1% (44.0 – 69.3%)	0.29	28	6	21.4% (4.5 – 61.3%)	0.48	
**School Attendance[4]**									
Unenrolled/poor	221	48	21.7% (8.9 – 44.0%)		226	44	19.5% (5.9 – 48.2%)		
Good attendance	1,177	930	79.0% (68.4 – 86.8%)	**<0.01**	1,062	470	44.3% (29.8 – 59.7%)	**0.03**	
**School Type**									
Private, boarding, other	378	290	76.7% (62.9 – 86.5%)		236	79	33.5% (17.3 – 54.8%)		
Public	796	636	79.9% (67.5 – 88.4%)	0.61	824	375	45.5% (29.2 – 62.8%)	0.29	
				District: Egor[5]					
Egor Total	27	27	100% (n/a)		130	86	66.2% (56.1 – 74.9%)		**<0.01**

	Pre-Mainstreaming (2021)				Post-Mainstreaming (2022)				
	SAC (5-14 years) (n)	*Took PZQ during MDA (n)*	*Weighted coverage estimate (95% CI)*	*p-value[1]*	*SAC (5-14 years) (n)*	*Took PZQ during MDA (n)*	*Weighted coverage estimate (95% CI)*	*p-value[1]*	*Pre- vs. Post- p-value[2]*
**Gender**									
Male	16	16	100% (n/a)		57	43	75.4% (69.7 – 80.4%)		
Female	11	11	100% (n/a)	n/a	73	43	58.9% (47.9 – 69.1%)	**<0.01**	
**Wealth Quintile[3]**									
Lowest 20%	0	n/a	n/a		0	n/a	n/a		
>20-40%	0	n/a	n/a		0	n/a	n/a		
>40-60%	11	11	100% (n/a)		0	n/a	n/a		
>60-80%	10	10	100% (n/a)		85	55	64.7% (49.3 – 77.6%)		
Top 20%	6	6	100% (n/a)	n/a	45	31	68.9% (62.5 – 74.7%)	0.72	
**School Attendance[4]**									
Unenrolled/poor	2	2	100% (n/a)		0	n/a	n/a		
Good attendance	25	25	100% (n/a)	n/a	130	86	66.2% (56.1 – 74.9%)	n/a	
**School Type**									
Private, boarding, other	17	17	100% (n/a)		115	73	63.5% (60.8 – 66.1%)		
Public	10	10	100% (n/a)	n/a	10	10	100% (n/a)	n/a	
District: Ughelli South[6]									
Ughelli South Total	123	107	87.0% (73.4 – 94.2%)		49	47	95.9% (n/a)		0.08
**Gender**									
Male	62	51	82.3% (68.0 – 91.0%)		21	21	100% (n/a)		
Female	61	56	91.8% (79.1 – 97.1%)	**0.001**	28	26	92.9% (n/a)	n/a	
**Wealth Quintile[3]**									
Lowest 20%	61	50	82.0% (67.1 – 91.0%)		5	5	100% (n/a)		
>20-40%	44	40	90.9% (76.4 – 96.9%)		24	23	95.8% (n/a)		

	Pre-Mainstreaming (2021)				Post-Mainstreaming (2022)				
	SAC (5-14 years) (n)	*Took PZQ during MDA (n)*	*Weighted coverage estimate (95% CI)*	*p-value[1]*	*SAC (5-14 years) (n)*	*Took PZQ during MDA (n)*	*Weighted coverage estimate (95% CI)*	*p-value[1]*	*Pre- vs. Post- p-value[2]*
>40-60%	16	16	100% (n/a)		19	18	94.8% (n/a)		
>60-80%	1	0	0% (n/a)		1	1	100% (n/a)		
Top 20%	1	1	100% (n/a)	0.14	0	n/a	n/a	n/a	
**School Attendance[4]**									
Unenrolled/poor	4	2	50.0% (16.5 – 83.5%)		1	0	0% (n/a)		
Good attendance	119	105	88.2% (74.7 – 95.0%)	**0.03**	48	47	97.9% (n/a)	n/a	
**School Type**									
Private, boarding, other	23	14	60.9% (27.9 – 86.2%)		23	22	95.7% (n/a)		
Public	99	93	93.9% (90.6 – 96.1%)	0.06	24	24	100% (n/a)	n/a	
District: Wamba									
Wamba Total	1,037	744	71.7% (53.7 – 84.8%)		910	611	67.1% (59.0 – 74.4%)		0.61
**Gender**									
Male	522	362	69.3% (48.9 – 84.3%)		491	324	66.0% (57.2 – 73.8%)		
Female	515	382	74.2% (58.3 – 85.5%)	0.19	419	287	68.5% (60.1 – 75.8%)	0.33	
**Wealth Quintile[3]**									
Lowest 20%	171	118	69.0% (39.5 – 88.4%)		186	109	58.6% (44.9 – 71.1%)		
>20-40%	172	122	70.9% (45.8 – 87.6%)		211	156	73.9% (55.7 – 86.5%)		
>40-60%	273	191	70.0% (49.2 – 84.9%)		238	153	64.3% (54.9 – 72.7%)		
>60-80%	320	226	70.6% (47.8 – 86.3%)		214	156	72.9% (66.2 – 78.7%)		
Top 20%	100	86	86.0% (61.0 – 96.0%)	0.60	53	34	64.2% (59.0 – 69.0%)	0.39	
**School Attendance[4]**									
Unenrolled/poor	17	5	29.4% (11.9 – 56.2%)		82	30	36.6% (20.1 – 57.0%)		

	Pre-Mainstreaming (2021)				Post-Mainstreaming (2022)				
	SAC (5-14 years) (n)	*Took PZQ during MDA (n)*	*Weighted coverage estimate (95% CI)*	*p-value[1]*	*SAC (5-14 years) (n)*	*Took PZQ during MDA (n)*	*Weighted coverage estimate (95% CI)*	*p-value[1]*	*Pre- vs. Post- p-value[2]*
Good attendance	1,011	732	72.4% (53.9 – 85.5%)	**0.001**	822	578	70.3% (61.4 – 77.9%)	**0.003**	
**School Type**									
Private, boarding, other	157	73	46.5% (35.8 – 57.5%)		212	157	74.1% (62.6 – 83.0%)		
Public	855	659	77.1% (55.0 – 90.2%)	**0.002**	603	411	68.2% (57.9 – 76.9%)	0.32	
Total for all districts									
Total	2,592	1,860	72.5% (62.6 – 80.6%)		2,382	1,259	55.4% (46.9 – 63.5%)		**<0.01**
**Gender**									
Male	1,323	943	71.7% (60.9 – 80.5%)		1,242	664	55.8% (47.1 – 64.3%)		
Female	1,269	917	73.4% (64.1 – 81.0%)	0.37	1,140	595	54.8% (46.3 – 63.2%)	0.64	
**Wealth Quintile[3]**									
Lowest 20%	591	440	74.8% (62.8 – 83.8%)		735	288	41.0% (27.1 – 56.5%)		
>20-40%	715	500	71.2% (56.0 – 82.8%)		603	348	60.7% (46.1 – 73.5%)		
>40-60%	580	425	73.7% (60.3 – 83.8%)		479	279	60.3% (49.7 – 70.1%)		
>60-80%	507	348	69.0% (52.9 – 81.5%)		412	265	65.5% (54.0 – 75.5%)		
Top 20%	191	141	75.8% (56.2 – 88.5%)	0.81	126	71	58.5% (46.3 – 69.7%)	**0.03**	
**School Attendance[4]**									
Unenrolled/poor	244	57	24.2% (11.5 – 43.8%)		309	74	24.8% (12.1 – 44.2%)		
Good attendance	2,332	1,792	77.0% (67.1 – 84.6%)	**<0.01**	2,062	1,181	59.7% (51.0 – 67.8%)	**<0.01**	
**School Type**									
Private, boarding, other	575	394	67.0% (56.0 – 76.3%)		586	331	59.9% (49.6 – 69.3%)		
Public	1,760	1,398	79.9% (67.8 – 88.3%)	**0.045**	1,461	820	58.2% (47.9 – 67.9%)	0.79	

	Pre-Mainstreaming (2021)				Post-Mainstreaming (2022)				
	SAC (5-14 years) (n)	*Took PZQ during MDA (n)*	*Weighted coverage estimate (95% CI)*	*p-value[1]*	*SAC (5-14 years) (n)*	*Took PZQ during MDA (n)*	*Weighted coverage estimate (95% CI)*	*p-value[1]*	*Pre- vs. Post- p-value[2]*

Abbreviations: MDA = Mass drug administration; n/a = Not applicable; PZQ = Praziquantel; SAC = School-aged children, 5-14 years

Bolded p-values are those that are <0.05.

1. p-value testing the difference between proportions of coverage by category of gender, wealth quintile, or school attendance within a single round of the survey.
2. p-value testing the difference between proportions in the pre- and post-mainstreaming coverage estimates for the sub-population of interest.
3. Wealth index variables were calculated for each household based on the methods adopted by the Demographic Health Survey (DHS) See Methods in text for details. Quintiles were defined separately for the pre-mainstreaming population and the post-mainstreaming population. The household wealth information was missing for 8 SAC in the pre-mainstreaming round, of whom 6 took PZQ, and for 27 children in the post-mainstreaming round, of whom 8 took PZQ. The p-value from this analysis comes from the test of wealth category as the sole predictor in a logistic regression incorporating survey weights, in order to test for a significant trend with increasing wealth quintile.
4. Attendance was classified as "Good" if the student reported attending school 'always' or 'most of the time' and as "Poor" if the student reported attending school "Part-time", "Sometimes", "Rarely", or "Never". The categories of 'Not enrolled' and 'Poor attendance' were collapsed for analysis due to small numbers within districts. The variable was missing for 16 SAC in the pre-mainstreaming round, of which 11 took PZQ, and for 11 SAC in the post-mainstreaming round, of which 4 took PZQ.
5. The pre-mainstreaming (2021) survey in Egor included only one community that was targeted for PZQ MDA, so there was no variance in Egor pre-mainstreaming coverage estimates, and no confidence intervals could be reported.
6. The post-mainstreaming (2022) survey in Ughelli South included only one community that was targeted for PZQ MDA, so there was no variance in Ughelli South post-mainstreaming coverage estimates, and no confidence intervals could be reported.
